# *Pneumocystis* Pneumonia Severity Is Associated with Taxonomic Shifts in the Respiratory Microbiota

**DOI:** 10.3390/pathogens14010082

**Published:** 2025-01-16

**Authors:** Valentina Del Prete, Antonia Piazzesi, Matteo Scanu, Francesca Toto, Stefania Pane, Federica Berrilli, Giovangiacinto Paterno, Lorenza Putignani, David di Cave

**Affiliations:** 1 Department of Clinical Sciences and Translational Medicine, University of Rome Tor Vergata, 00133 Rome, Italy; valentina.delprete90@libero.it (V.D.P.); federica.berrilli@uniroma2.it (F.B.); 2 Management and Diagnostic Innovations & Clinical Pathways Research Area, Unit of Microbiome, Bambino Gesù Children’s Hospital, IRCCS, 00144 Rome, Italy; afelicia.piazzesi@opbg.net (A.P.); matteo.scanu@opbg.net (M.S.); francesca.toto@opbg.net (F.T.); 3 Unit of Microbiology and Diagnostic Immunology, Unit of Microbiomics, Bambino Gesù Children’s Hospital, IRCCS, 00144 Rome, Italy; stefania.pane@opbg.net; 4 Hematology, Department of Biomedicine and Prevention, University of Rome Tor Vergata, 00133 Rome, Italy; giovangiacinto.paterno@ptvonline.it; 5 Unit of Microbiology and Diagnostic Immunology, Unit of Microbiomics and Management and Diagnostic Innovations & Clinical Pathways Research Area, Unit of Microbiome, Bambino Gesù Children’s Hospital, IRCCS, 00144 Rome, Italy

**Keywords:** respiratory microbiota, *Pneumocystis jirocevii*, *Pneumocystis* pneumonia, metataxonomy sequencing, lung

## Abstract

Pneumonia caused by *Pneumocystis jirovecii* infection (PCP) is a potentially life-threatening illness, particularly affecting the immunocompromised. The past two decades have shown an increase in PCP incidence; however, the underlying factors that promote disease severity and fatality have yet to be fully elucidated. Recent evidence suggests that the microbiota of the respiratory tract may play a role in stimulating or repressing pulmonary inflammation, as well as the progression of both bacterial and viral pneumonia. Here, we employed 16S rRNA metataxonomic sequencing to profile the respiratory microbiota of patients with mild-moderate and severe PCP. Our results show that the upper and lower airways of PCP patients have bacterial profiles which have been associated with a pro-inflammatory response. Furthermore, we find that severe PCP is associated with lower bacterial diversity and an increase in *Prevotella* and a decrease in *Neisseria*. Functionally, severe PCP was associated with a decrease in metabolic pathways of molecules with anti-inflammatory and antimicrobial properties. To our knowledge, this is the first study showing an association of PCP severity with shifts in the respiratory microbiome and may provide some insight into which patients are more susceptible to the more severe manifestations of the disease.

## 1. Introduction

*Pneumocystis jirovecii*, an opportunistic fungal pathogen, is the causative agent of *Pneumocystis* pneumonia (PCP), a life-threatening interstitial pneumonia that disproportionately affects immunocompromised individuals [1,2]. This includes those infected with the human immunodeficiency virus (HIV), cancer patients undergoing chemotherapy (particularly patients with hematological malignancies), organ and stem cell transplantation recipients, individuals with primary immunodeficiencies, or patients undergoing corticosteroid or lymphodepleting treatments [3,4,5,6,7,8,9]. *P. jirovecii* demonstrates a specific affinity for pulmonary epithelial cells, allowing it to reside within the alveoli in an extracellular form and draw sustenance from alveolar fluids. The immune response against *P. jirovecii* is initiated by tumor necrosis factor α (TNF-α) and interleukin-1 (IL-1), produced by alveolar macrophages that identify the pathogen’s glycoprotein membrane, facilitated by opsonizing IgGs. This process leads to phagocytosis of the microorganism and triggers an inflammatory response mediated by T-helper cells (CD4+) and interferon gamma, aimed at clearing *P. jirovecii*; however, it may also result in immune-mediated lung damage [10].

In the late 90s, there was a sharp decline in PCP incidence among HIV-infected individuals, largely due to the introduction of prophylactic therapies, as well as the antiretroviral therapies which decreased the incidence of AIDS and thus AIDS-defining infections such as PCP [2,11,12]. However, recent studies have found that the incidence of PCP is rising again across Europe, particularly in the non-HIV population [13]. While factors such as age, co-infections, and the use of invasive ventilation during hospitalization have all been associated with PCP mortality to varying degrees [14,15], what underlying factors create a permissive environment for *P. jirovecii* to thrive and overcome the patients still remain to be fully elucidated.

The lungs, once considered a sterile environment, are now recognized to harbor a diverse microbiome comprising bacteria, fungi, and viruses, thanks to recent advances in next-generation sequencing [16,17,18]. This microbiome of the respiratory tract plays a crucial role in maintaining lung health and can be significantly altered during disease states [19]. The interplay between these microbial communities and lung pathogens like *P. jirovecii* is an area of active research, with recent studies suggesting a potential role of the microbiome in modulating disease severity [20,21,22].

Studies have shown that the commensal microbiota of the respiratory tract can influence pathogen colonization, competition, and virulence [23,24,25,26]. Furthermore, specific microbial patterns may either enhance immune defense mechanisms or exacerbate inflammatory responses, impacting disease severity and progression [27,28]. Therefore, profiling the respiratory microbiome in PCP patients can help identify microbial biomarkers associated with disease severity and response to treatment.

## 2. Materials and Methods

### 2.1. Patient Enrollment

Patients were enrolled between April 2020 and December 2021. Patients over 18 years of age, admitted to the Fondazione Policlinico Tor Vergata in Rome for PCP, and who agreed to participate in the study were enrolled. Wherever possible, bronchoalveolar lavage (BAL) samples were collected from patients, as well as information regarding age, gender, underlying medical conditions, disease severity, and pulmonary co-infections (Table 1). Those patients who were unable to provide BAL samples due to their underlying medical conditions provided sputum (SPT) samples. The study was approved by the local institutional review board on the 24th of November 2021 (Pro RS 237/21). At the time of biological sample collection, all patients were receiving empirical antibiotic therapy, while 7 patients were receiving prophylactic therapy for PCP with trimethoprim-sulfamethoxazole. The anti-infective therapy was subsequently modulated based on the results of the microbiological findings. All patients provided informed consent for the processing of their sensitive data, and all clinical data were analyzed anonymously.

### 2.2. P. jirovecii Diagnosis

All samples were processed with MeriFluor *Pneumocystis* (Meridian Bioscience, Cincinnati, OH, USA) for immunofluorescence (IFA) and quantified by an expert microbiologist. *P. jirovecii* infection was confirmed when the fungus was identified after microscopic examination of BAL or SPT samples revealed five or more fluorescent asci on a slide. Infection was further confirmed by real-time (RT)-PCR.

### 2.3. PCP Severity Scoring

Patients with confirmed PCP were categorized as mild, moderate, or severe based on objective clinical parameters [11]. Patients requiring no supplemental oxygen were categorized as having mild PCP. Patients requiring supplemental oxygen, with a respiration rate between 25 and 30 breaths/min, and/or a decreased blood pressure of under 90 mm Hg systolic and over 50 mm Hg diastolic were categorized as having moderate PCP. Patients in hypoxemic acute respiratory failure requiring high-flow nasal oxygen with at least 50% FiO_2_, non-invasive ventilation, or mechanical ventilation, a respiration rate over 30 breaths/min, and/or a decreased blood pressure of under 90 mm Hg systolic and over 50 mm Hg diastolic were categorized as having severe PCP.

### 2.4. P. jirovecii RT-PCR

DNA extraction was performed from biological samples using a commercially available kit (QIAamp DNA kit; QIAGEN, Hilden, Germany) according to the manufacturer’s instructions on an EZ-1 Advanced XL automated extractor. *P. jirovecii* DNA was amplified using the RealCycler PJIR kit (Progenie Molecular, Valencia, Spain) [29,30]. The detection of *P. jirovecii* in the examined samples was based on PCR amplification of a fragment of the gene encoding the large subunit region of mitochondrial ribosomal DNA (mtLSU-rRNA). All amplification reactions included a negative control (to exclude potential contamination during processing) and a positive control (to test reagent validity). The amplification process involved two PCR cycles [31]. The first PCR cycle amplified a 346 bp fragment using primers pAZ102-E and pAZ102-H79 in an endpoint PCR:

pAZ102E (forward primer): 5′-GATGGCTGTTTCCAAGCCCA-3′

pAZ102H (reverse primer): 5′-GTGTACGTTGCAAAGTACTC-3′

The second PCR cycle utilized a nested PCR protocol applied to all samples for the amplification of a 260 bp fragment using primers pAZ102-X and pAZ102-Y79:

pAZ102X: 5′-GTGAAATACAAATCGGACTAGG-3′

pAZ102Y: 5′-TCACTTAATATTAATTGGGGAGC-3′

Both PCR cycles were performed using the T Professional Basic Gradient thermocycler (BIOMETRA, Göttingen, Germany). The PCR-amplified samples were prepared for sequencing by purification with ExoSap-IT (Express PCR Product Cleanup, Merck, Darmstadt, Germany). This kit is used for direct purification of PCR products and DNA fragments on 2% agarose gels. This protocol generates high-quality sequences using a BigDye™ Cycle Terminator v3.1 sequencing kit (Merck, Darmstadt, Germany) and the Applied Biosystems™ genetic analyzer (Thermofisher Scientific, Waltham, MA, USA).

### 2.5. 16S rDNA Sequencing

The V3–V4 hypervariable region of the bacterial 16S rRNA gene was amplified as per the MiSeq rRNA Amplicon Sequencing workflow (Illumina, San Diego, CA, USA). Amplicons were purified with AMPure XP beads (Beckman Coulter Inc., Brea, CA, USA) and indexed with Nextera XT indexes (Illumina, San Diego, CA, USA) as per the 16S Metagenomic Sequencing Library Preparation protocol (Illumina, San Diego, CA, USA). Final DNA concentrations were obtained with the PicoGreen Assay for dsDNA (ThermoFisher Scientific, Waltham, MA, USA). Samples were then pooled, diluted, and denatured as per the MiSeq System Denature and Dilute Libraries Guide (Illumina, San Diego, CA, USA) and sequenced on a MiSeqDx sequencer (Illumina, San Diego, CA, USA).

### 2.6. Metataxonomic and Functional Analysis

Paired-end sequences were imported into CLC Genomics Workbench (Qiagen, v23.0.4, Hilden, Germany) with sequencing primers trimmed, and low-quality sequences were discarded. Taxonomic profiling was conducted with the Microbial Genomics Module in CLC Genomics Workbench interrogating the Greengenes database. Differential abundance analysis was conducted with the Microbial Genomics Module using a negative binomial generalized linear model (GLM) corrected for FDR. PiCRUST analysis was performed with the microbial genomics module using an EC terms multiplication table, and pathways were identified by interrogating the MetaCyc database. All databases were downloaded within the CLC Genomics Module and interrogated with the software’s own algorithms.

### 2.7. Statistical Analysis

Statistically significant differences in the relative abundances of bacterial taxa were determined with CLC Genomics Workbench (Qiagen, Hilden, Germany) with a negative binomial generalized linear model (GLM). Multiple comparisons were corrected for with false discovery rate (FDR). Statistically significant differences in alpha diversity were determined in GraphPad Prism v. 8.0.2 (Boston, MA, USA) with a Kruskal–Wallis test.

## 3. Results

### 3.1. Upper and Lower Airways Have a Similar Community Structure in Patients with PCP

In total, 33 patients were recruited to participate in this observational, monocentric study. Due to their clinical condition, only 24 of these were able to provide BAL samples, while the other 9 provided sputum (SPT) for metataxonomic analysis (Table 1).

**Figure 1:**
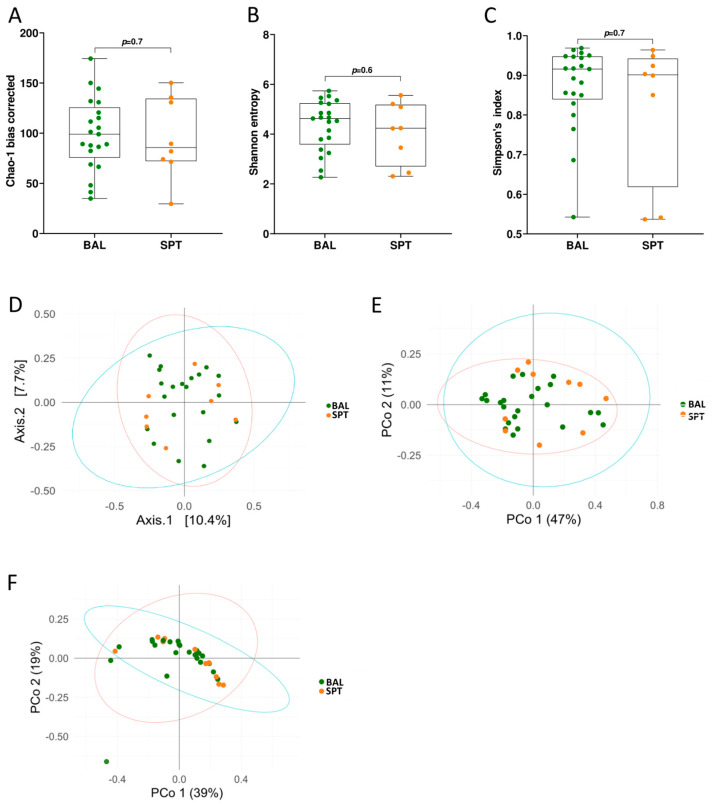
Bacterial diversity is similar between upper and lower airways. (**A**–**C**) Bacterial alpha diversity measurements in bronchoalveolar lavage (BAL) and sputum (SPT) samples in patients with *Pneumocystis* pneumonia (PCP), measured by bias-corrected Chao-1 (**A**), Shannon entropy (**B**), and Simpson’s index (**C**) analyses. Statistical analysis: Kruskal–Wallis test. (**D**–**F**) Beta-diversity analysis as measured by Bray–Curtis dissimilarity analysis (**D**), weighted UniFrac (**E**), and unweighted UniFrac (**F**) analyses on bacterial populations in BAL and SPT samples from patients with PCP.

In healthy individuals, there are significant differences between the microbial composition of the upper and lower airways, with the upper airways being associated with higher bacterial load and being overpopulated with specific, supraglottic-specific taxa [32]. However, differences in the ecological composition between these two sites have never been addressed in the context of *P. jirovecii* infection. Therefore, we first decided to compare these two sample types with each other to see whether there were significant differences between the ecological composition between the upper and lower airways. To do this, we first measured bacterial diversity by alpha- and beta-diversity methods (Figure 1). We found that microbial richness was comparable between the two sites, as measured by bias-corrected Chao-1 (Figure 1A), Shannon entropy (Figure 1B), and Simpson’s index (Figure 1C) methods. Interestingly, the two groups also did not cluster separately when analyzed with Bray–Curtis dissimilarity (Figure 1D), or with weighted or unweighted UniFrac measurements (Figure 1E,F), indicating that the microbial communities in the upper and lower airways are similar in patients with PCP.

**Figure 2:**
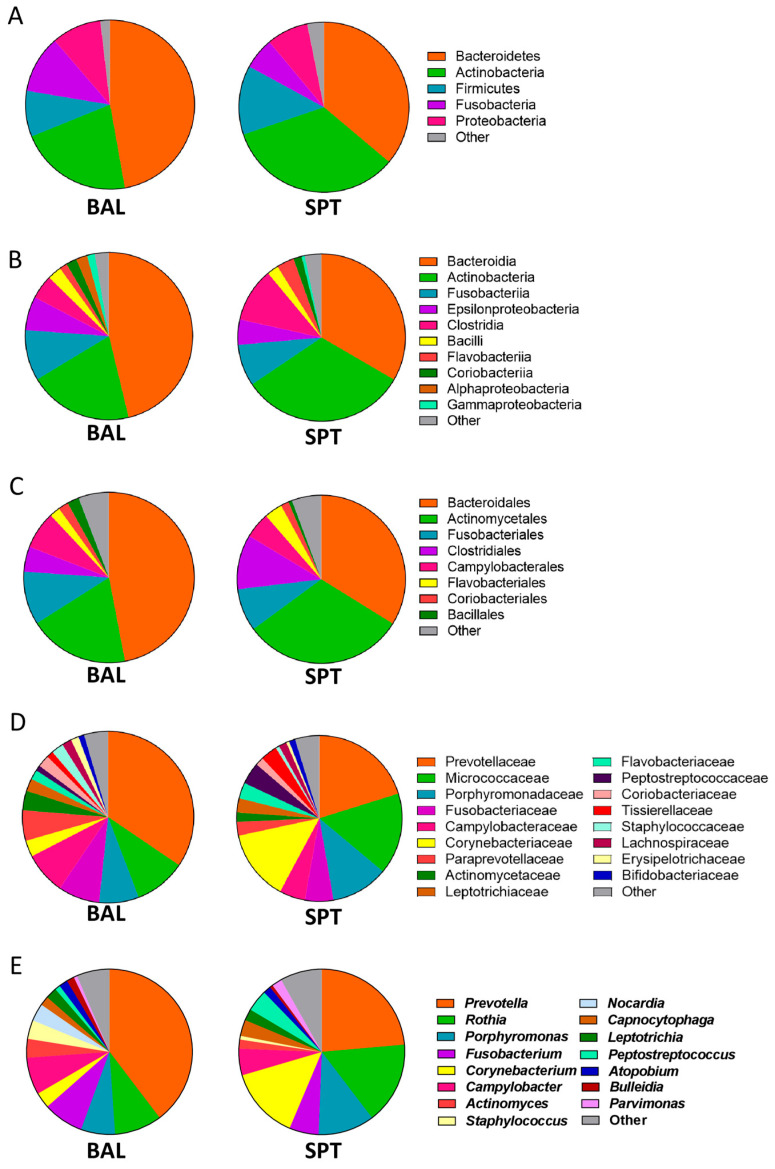
Metataxonomy of bacterial populations in the upper and lower airways of patients with PCP. (**A**–**E**) Pie charts of the most abundant phyla (**A**), classes (**B**), order (**C**), families (**D**), and genera (**E**) in BAL and SPT samples in patients with PCP.

In both ecological niches, Bacteroidetes was the dominant phylum, representing, on average, over 40% of the total bacterial community (Figure 2A). The second most abundant phylum on average was Actinobacteria, followed by Firmicutes, Fusobacteria, and Proteobacteria (Figure 2A). The remaining 1.8–3.2% was composed of up to 10 different phyla found in very low abundances across different patients, including Tenericutes, Synergistetes, and Chloroflexi.

Within the Bacteroidetes phylum, the Bacteroidia class and the Bacteroidales order predominated (Figure 2B,C). This order was largely composed of the Prevotellaceae family, followed by the Porphyromonadaceae family (Figure 2D), which in turn were composed of the *Prevotella* and *Porphyromonas* genera, respectively (Figure 2E). Within the Actinobacteria phylum, the Actinobacteria class and the Actinomycetales order predominated, which was found to be largely composed of the *Rothia*, *Corynebacterium*, and *Actinomyces* genera (Figure 2A–E). Other abundant genera found in these samples were *Fusobacterium*, *Campylobacter*, *Leptotrichia*, *Capnocytophaga*, and *Peptostreptococcus* (Figure 2E).

### 3.2. Subtle Taxonomic Shifts in Bacterial Communities Between Upper and Lower Airways

**Figure 3:**
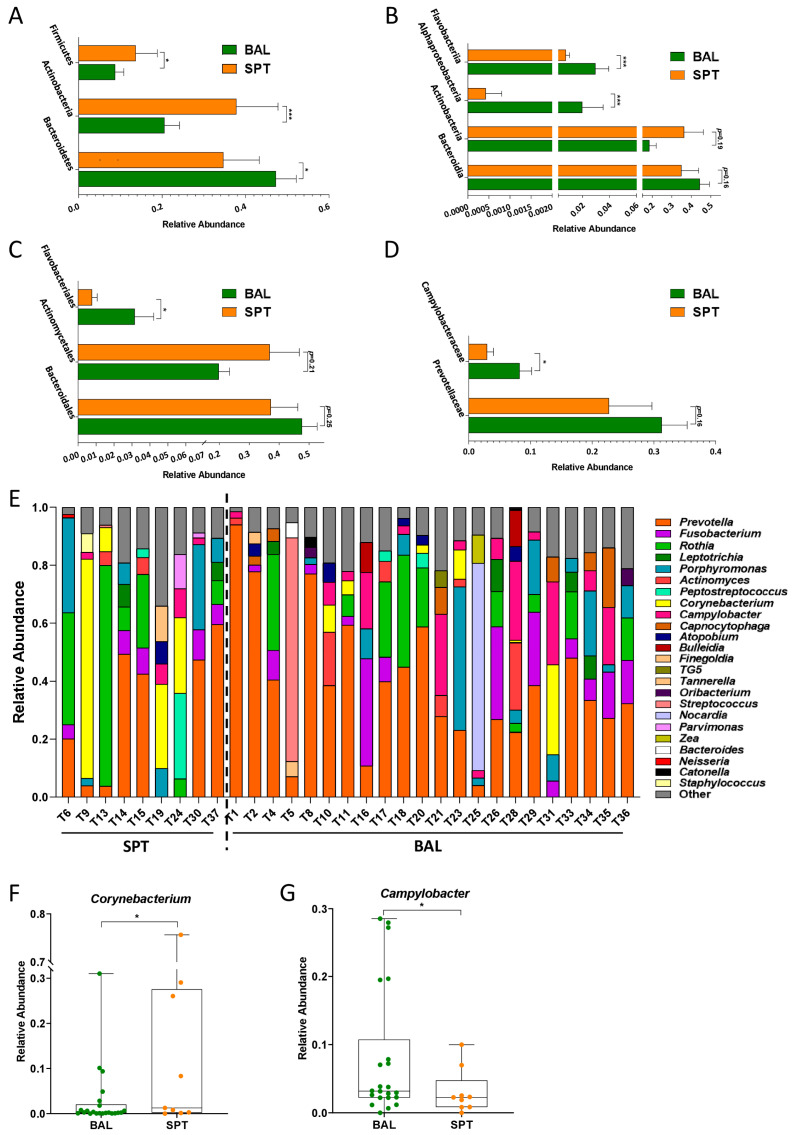
Differentially abundant bacterial taxa in patients with PCP. (**A**–**D**) Bar charts of differentially abundant phyla (**A**), classes (**B**), orders (**C**), and families (**D**) in BAL samples (green) compared to SPT (orange). All bars represent mean ± S.E.M. (**E**) Bar chart of the most abundant genera in each patient sample. (**F**,**G**) Boxplots of the relative abundances in Corynebacterium (**F**) and Campylobacter (**G**) in BAL and SPT samples in patients with PCP. Statistical analysis: negative binomial generalized linear model (GLM) corrected for FDR. * *p* < 0.05, *** *p* < 0.001.

Despite having similar microbial community structures, subtle differences in the relative abundances of certain taxa were observed between SPT and BAL samples in these patients. Specifically, the lower airways were characterized by a significant increase in the Bacteroidetes phylum (Figure 3A), mostly due to an increased abundance in the Flavobacteriia class and the Flavobacteriales order, as well as a slight but non-significant increase in Bacteroidia and Bacteroidales (Figure 3B,C). Furthermore, though the Proteobacteria phylum was not significantly altered as a whole, BAL samples from the lower airways were also significantly enriched in the Alphaproteobacteria class and the Campylobacteriaceae family (Figure 3B,D).

SPT sampled from the upper airways, on the other hand, were characterized by an increase in Actinobacteria and Firmicutes phyla (Figure 3A), followed by an increase in the relative abundance of the Actinobacteria class and Actinomyocetales order; however, these did not maintain statistical significance when corrections for multiple comparisons were applied (Figure 3B,C).

At the genus level, we found that the relative abundances of different genera varied substantially between different individual patients, which could explain, in part, why the two groups did not form distinct clusters despite there being statistically significant shifts in some taxa (Figure 3E). However, despite this variability, we found that BAL samples were characterized by a significant increase in the *Campylobacter* genus, while the significant increase in the Actinobacteria phylum observed in SPT samples was due in part to a significant increase in *Corynebacterium* (Figure 3F,G).

**Figure 4:**
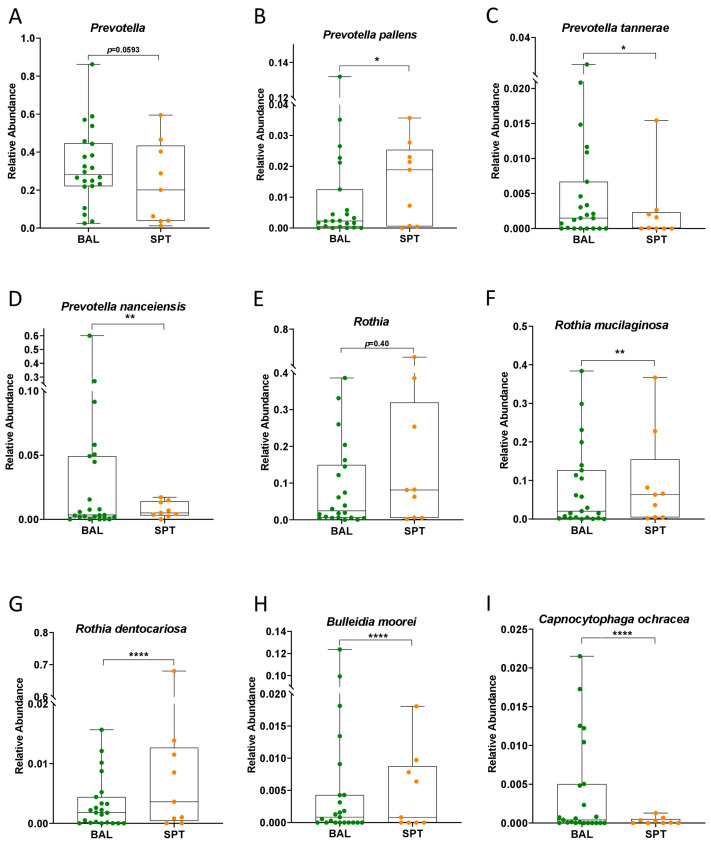
Differentially abundant bacterial species in patients with PCP. (**A**–**I**) Boxplots of the relative abundances in *Prevotella* (**A**), *Prevotella pallens* (**B**), *Prevotella tannerae* (**C**), *Prevotella nanceiensis* (**D**), *Rothia* (**E**), *Rothia mucilaginosa* (**F**), *Rothia dentocariosa* (**G**), *Bulleidia moorei* (**H**), and *Capnocytophaga ochracea* (**I**) in BAL and SPT samples in patients with PCP. Statistical analysis: negative binomial generalized linear model (GLM) corrected for FDR, * *p* < 0.05; ** *p* < 0.01; **** *p* < 0.0001.

The observed increase in Bacteroidetes in BAL samples was largely due to an increase in Prevotella; however, this too did not maintain statistical significance when corrections for multiple comparisons were applied (Figure 4A). However, we also observed that BAL samples were significantly enriched in the species *Prevotella tannerae* and *Prevotella nanceiensis*, while *Prevotella pallens* was, on average, more enriched in SPT samples (Figure 4B–D). Furthermore, the increase observed in the Actinobacteria phylum in SPT samples was due in part to a significant enrichment of *Corynebacterium*, but also of *Rothia mucilaginosa* and *Rothia dentocariosa*; however, the *Rothia* genus itself was not found to be significantly increased (Figure 4E–G). Finally, BAL samples were also significantly enriched in *Bulleidia moorei* and *Capnocytophaga ochracea*, the latter being mostly responsible for the observed significant increase in the Flavobacteriia class (Figure 4H,I).

### 3.3. PCP Severity Is Associated with Reduced Bacterial Diversity

In order to see whether pneumonia severity brought about a larger shift in microbial richness than body site, we next stratified patients by PCP severity, focusing exclusively on BAL samples (Table 2).

**Figure 5:**
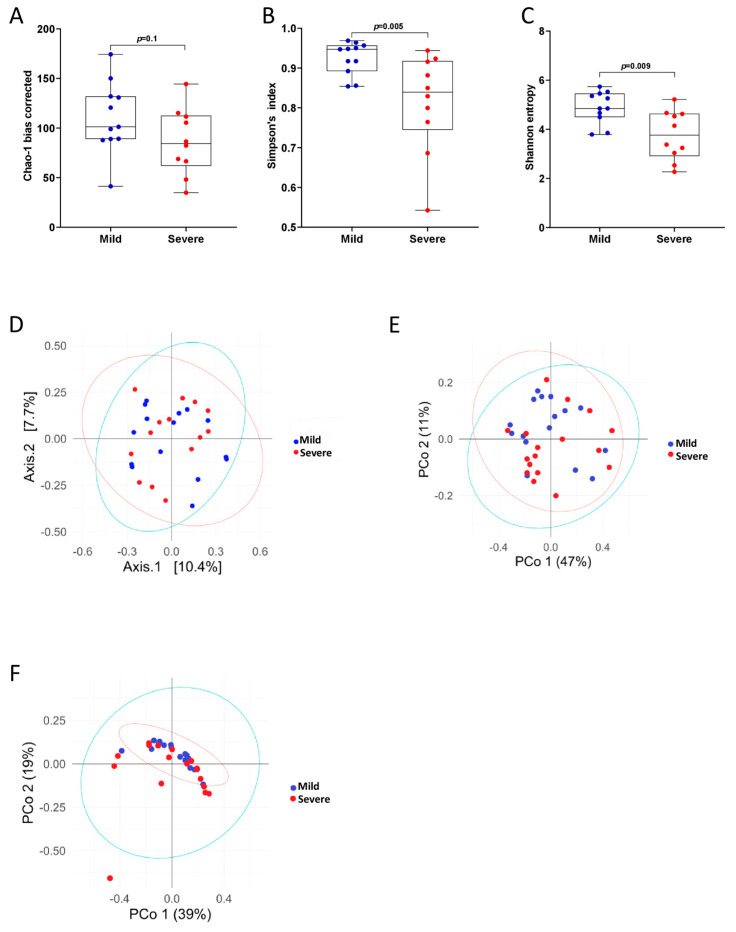
Patients with severe PCP have lower bacterial diversity than patients with mild-moderate PCP. (**A**–**C**) Bacterial alpha diversity measurements in BAL samples in patients with mild-moderate and severe PCP, measured by bias-corrected Chao-1 (**A**), Shannon entropy (**B**), and Simpson’s index (**C**) analyses. Statistical analysis: Kruskal–Wallis test. (**D**–**F**) Beta-diversity analysis as measured by Bray–Curtis dissimilarity analysis (**D**), weighted UniFrac (**E**), and unweighted UniFrac (**F**) analyses on bacterial populations in BAL samples from patients with mild-moderate and severe PCP.

We found that increasing PCP severity led to an overall decrease in bacterial alpha-diversity (Figure 5A–C). While this decreasing trend was not statistically significant when measured by bias-corrected Chao-1 methods (Figure 5A), patients with severe PCP did have significantly reduced bacterial diversity when compared to patients with mild-moderate PCP when measured by Shannon entropy (Figure 5B) and Simpson’s index (Figure 5C). However, as observed when comparing BAL and SPT samples, the microbiota of the lower airways of patients with mild-moderate and severe PCP failed to form statistically significant separate clusters when analyzed with Bray–Curtis dissimilarity (Figure 5D), or with weighted or unweighted UniFrac methods (Figure 5E,F).

### 3.4. Taxonomic Shifts in the Microbiota of the Lower Airways Are Observed in Severe PCP

**Figure 6:**
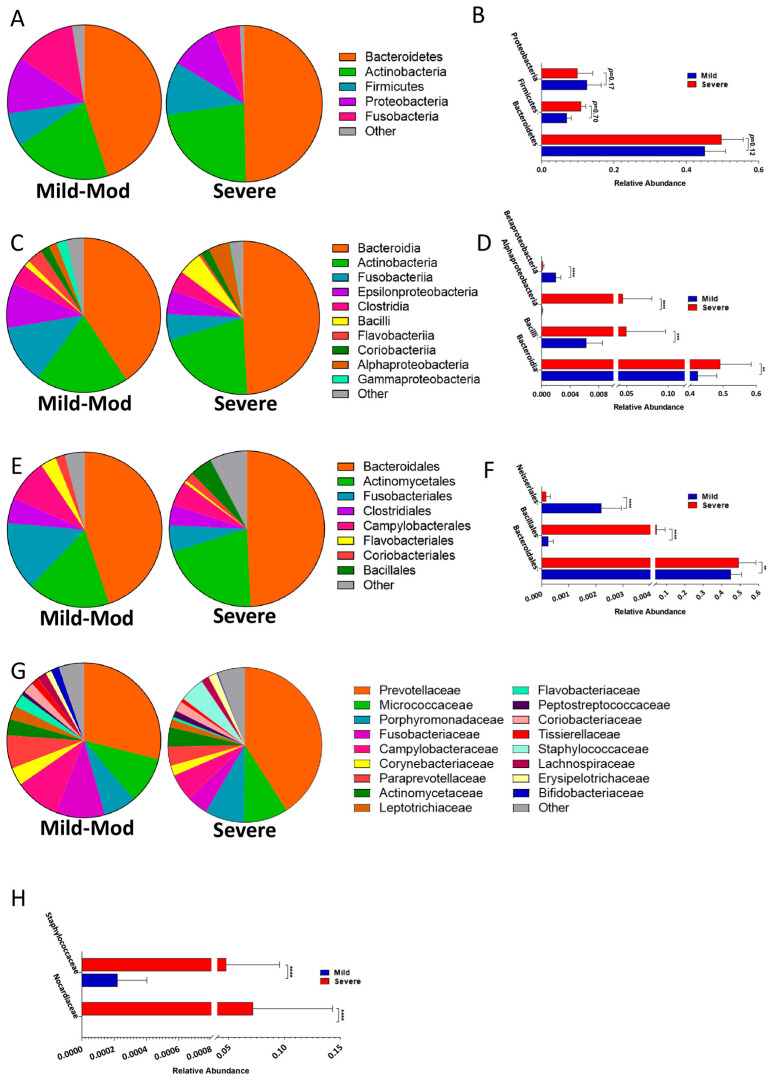
Metataxonomy of bacterial populations in the lower airways of patients with PCP. (**A**,**B**) Pie chart (**A**) and bar plot (**B**) of the most abundant phyla in BAL samples of patients with PCP. (**C**,**D**) Pie chart (**C**) and bar plot (**D**) of the most abundant classes in BAL samples of patients with PCP. (**E**,**F**) Pie chart (**E**) and bar plot (**F**) of the most abundant orders in BAL samples of patients with PCP. (**G**,**H**) Pie chart (**G**) and bar plot (**H**) of the most abundant families in BAL samples of patients with PCP. Statistical analysis: negative binomial generalized linear model (GLM) corrected for FDR. ** *p* < 0.01; *** *p* < 0.001; **** *p* < 0.0001.

As observed when comparing microbial populations from upper and lower airways, severe PCP was also associated with shifts in their metataxonomy (Figure 6; Supplementary Table S1). While there were no significant differences observed at the phylum level (Figure 6A,B), patients with severe PCP were characterized by a significant enrichment of bacteria belonging to the Bacteroidia, Bacilli, and Alphaproteobacteria classes (Figure 6C,D). The increase in the Bacilli class was largely due to an enrichment in the Bacillales order and the Staphylococcaceae family, while the enrichment of Bacteroidia was largely due to an enrichment of Bacteroidales (Figure 6E–H).

**Figure 7:**
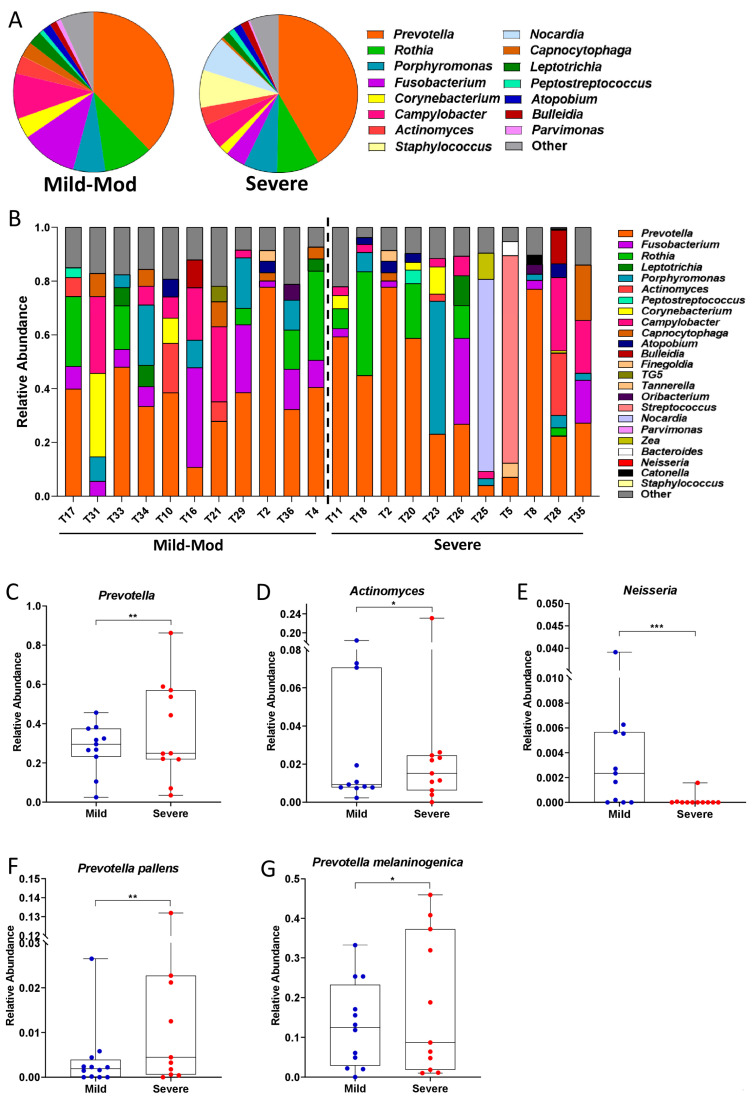
Metataxonomy of bacterial genera and species in the lower airways of patients with PCP. (**A**) Pie chart of the most abundant genera in BAL samples of patients with PCP. (**B**) Stacked bar plot of the most abundant genera in each patient with PCP. Genera under 1% relative abundance were collectively represented as “other”. (**C**–**G**) Boxplots of the relative abundances in *Prevotella* (**C**), *Actinomyces* (**D**), *Neisseria* (**E**), *Prevotella pallens* (**F**) and *Prevotella melaninogenica* (**G**), in BAL samples in patients with mild-moderate and severe PCP. Statistical analysis: negative binomial generalized linear model (GLM) corrected for FDR. * *p* < 0.05; ** *p* < 0.01; *** *p* < 0.001.

At the genus level, we can observe that the increase in Bacteroidales is likely due to the significant enrichment of *Prevotella* in patients with severe PCP (Figure 7A–C). Consistently, patients with severe PCP also had significant enrichment of *Prevotella pallens* and *Prevotella melaninogenica* in the microbiome of the lower respiratory tract, as well as an enrichment of the genus Actinomyces (Figure 7D,F,G).

Patients with mild-to-moderate PCP, on the other hand, were characterized by a significant enrichment in Betaproteobacteria (Figure 6C,D), which was largely due to the Neisseriales order (Figure 6E,F) and the Neisseria genus (Figure 7E). In fact, out of 11 patients with severe PCP, Neisseria was only detected in the BAL of 2 of them, while this genus was detected in 8 out of 11 patients with mild-moderate PCP (Figure 7E).

### 3.5. Severe PCP Is Associated with Altered Microbial Functional Pathways

Finally, we investigated which functional pathways were likely to be most affected by these shifts in bacterial taxa of the lower airways by performing Phylogenetic Investigation of Communities by Reconstruction of Unobserved States (PiCRUST) analysis.

**Figure 8:**
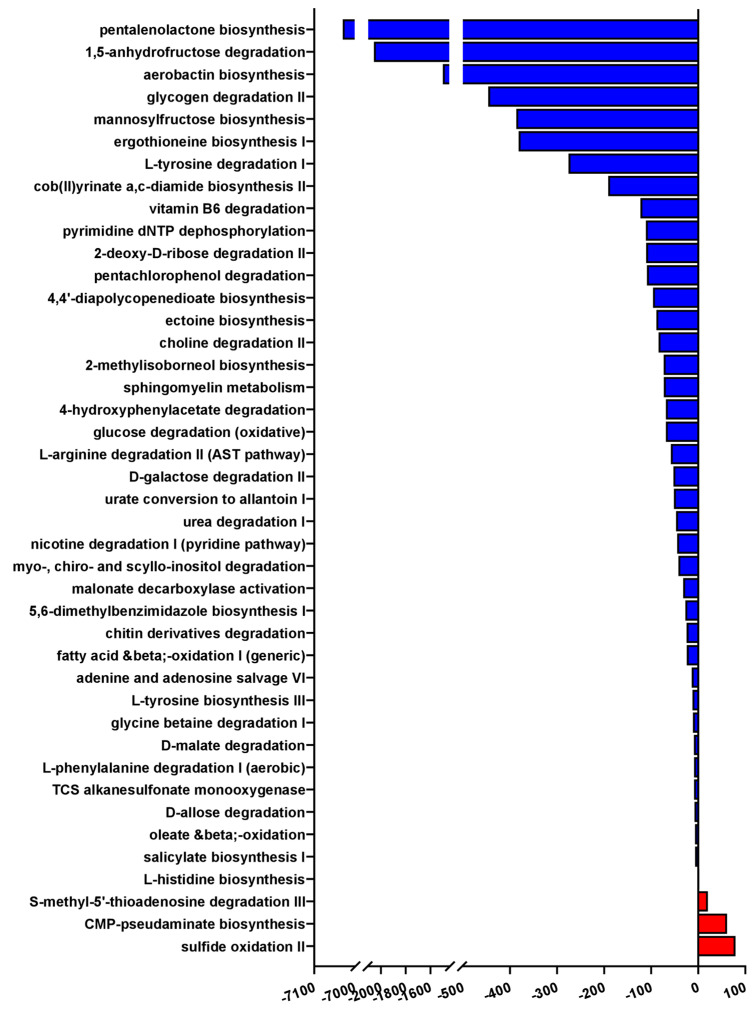
Inferred functional profiles of pulmonary bacterial populations in patients with mild-moderate versus severe PCP. Bar chart of microbial metabolic pathways predicted to be differentially regulated in the lower airways of patients with PCP. Each bar represents one metabolic pathway, only statistically significant results with a confidence cutoff of 1 are represented. Blue: pathways predicted to be overrepresented in patients with mild-moderate PCP; red: pathways predicted to be enriched in patients with severe PCP.

Using a confidence cut-off of 1, we found that 42 microbial functional pathways were predicted to be significantly enriched in patients with mild-to-moderate PCP compared to severely affected patients, with only 3 pathways being predicted to be activated in those with severe PCP (Figure 8). The most highly enriched pathway in the lungs was sulfide oxidation, i.e., the catabolism of hydrogen sulfide into sulfur, which itself has been associated with lung inflammation and respiratory diseases [20]. Similarly, patients with severe PCP were also found to have a predicted functional profile of enriched S-methyl-5′-thioadenosine degradation, a metabolite which has also been linked to prognosis in chronic obstructive pulmonary disease [19].

Far more functional pathways were predicted to be enriched in mild-moderately affected patients than severe ones, a finding which is consistent with the reduced bacterial diversity observed in patients with severe PCP (Figure 5A–C and Figure 8). The most overrepresented pathway by far is the biosynthesis of pentalenolactone, with an average abundance of over 7000-fold in patients with mild-moderate forms of the disease compared to those with severe PCP (Figure 8). Though not much is known about this metabolite in the context of pneumonia, pentalenolactone is a sesquiterpene lactone, a class of molecules with antibiotic activity which have been found, in in vitro studies, to also possess anti-inflammatory and anti-tumorigenic properties [33]. The second most overrepresented pathway in patients with mild-moderate PCP, with an over 2000-fold increase, is 1,5-anhydrofructose degradation, which is part of the glycogen catabolic process, and also highly relevant to a wide array of pulmonary diseases [34]. Furthermore, 1,5-anhydro-D-fructose is itself a molecule with anti-inflammatory and antimicrobial activity, as well as being a precursor to other antibiotics [35,36]. Taken together, these results suggest that, both taxonomically and functionally, the microbiome of the lower airways of patients with severe PCP possesses several pro-inflammatory signatures when compared to those with mild-moderate PCP.

## 4. Discussion

In this monocentric, observational study, we describe the ecological composition of the respiratory microbiota of patients with PCP. Though, to date, two other studies have investigated the microbial composition of the lower airways in patients with PCP [18,37], this is the first study to compare these two distinct body sites, as well as associate ecological shifts with the severity of the pneumonia.

As a subset of our patients were not able to undergo BAL sample collection due to their underlying condition and due to the relatively invasive nature of this technique, we first decided to compare metataxonomic composition of the lower airways with the upper airways in PCP patients. Contrary to what has been reported in healthy individuals [32], we did not find significant differences in bacterial diversity or global ecological composition between these two body sites in PCP patients (Figure 1). Indeed, only a few bacterial taxa, of which two bacterial genera, namely *Corynebacterium* and *Campylobacter*, were found to be significantly altered between BAL and SPT samples (Figure 3F,G). Interestingly, there were also species within the *Prevotella* genus, namely *P. tannerae* and *P. nanceinensis*, which were significantly enriched in BAL samples compared to SPT (Figure 4C,D). As *Prevotella* is a genus that has been associated with the upper airways [32], these results are also contradictory to what has been reported in healthy individuals. Taken together, these results suggest that both the upper and lower airways of PCP patients exhibit a distinct ecological profile.

Next, we decided to focus exclusively on BAL samples in order to describe the metataxonomic composition of patients affected with mild or severe forms of PCP. In line with our expectations and with previously published results [19,21,32], most samples were overpopulated with one bacterial genus, with the top 5 genera comprising 80–98% of the relative abundance of the bacterial population of the respiratory tract. In our patient cohort, only 3 out of 34 did not have *Prevotella* as one of their top 5 most abundant genera and, in 19 out of 34 patients (56%), *Prevotella* was the most abundant genus in the microbiota of the lower respiratory tract (Figure 3E). Interestingly, the *Corynebacterium* genus was abundant only in samples where *Prevotella* was either in very low abundance or absent from these top 5 genera and, conversely, was present in very low relative abundances or not at all where *Prevotella* dominated (Figure 3E). However, contrary to previously published studies, *Veillonella* was only found in very low relative abundances throughout this cohort, never appearing in the top 5 most abundant genera in any patient (Figure 2E and Figure 3E). Instead, high relative abundances of *Prevotella* were most often accompanied by the *Rothia*, *Fusobacterium*, and *Porphyromonas* genera, while *Corynebacterium* was most often found in conjunction with *Campylobacter* (Figure 3E). Taken together, these results indicate that, while there do appear to be two distinct pneumotypes in our patient cohort, the respiratory microbiome dominated by *Prevotella*, *Rothia*, and *Fusobacterium* is by far the most common among PCP patients.

Previous studies on the microbiome of the lower respiratory tract have indicated that the *Prevotella* genus is characteristic of the supraglottis, and that its presence in relatively high abundances in the lower airways can be due to either contamination during BAL sample collection or due to increased lung inflammation [32]. In this study, we found not only that *Prevotella* was enriched in the lower airways, but several *Prevotella* species were also significantly enriched in BAL samples compared to SPT (Figure 4A–D), making supraglottal contamination an unlikely cause of this finding. Furthermore, *Prevotella* was significantly enriched in patients with severe PCP compared to those with mild-moderate manifestations of the disease, consistent with the hypothesis that the increased *Prevotella* in the lungs is tied to increased inflammation rather than supraglottal contamination. Furthermore, PiCRUST analysis predicted a significant decrease in metabolic processes associated with anti-inflammatory and antimicrobial molecules in patients with severe PCP (Figure 8). Taken together, these results indicate that the microbiome of the lower airways of severely affected patients reflects the state of enhanced inflammation and disease from which they suffer.

Patients suffering from severe PCP were also found to have reduced populations of *Neisseria*. Indeed, we only detected this genus in 2 out of 11 severely affected patients, while it was detected in 8 out of 11 mild-moderately ill PCP patients (Figure 7E). Interestingly, a reduction of *Neisseria* in the lungs is associated with a reduction in alpha-diversity in patients with chronic *Pseudomonas aeruginosa* infection [19,38], consistent with what we observed in our severely ill patients (Figure 5B,C). On the other hand, other studies have found that increases of certain *Neisseria* species correlate positively with inflammation and poor prognosis in patients with bronchiectasis [39], which is inconsistent with the picture of increased pulmonary inflammation that the microbiome of the lower airways, when observed as a whole, seems to have painted in patients with severe PCP [40,41]. Further studies are needed to investigate which *Neisseria* species are overrepresented in the mild-moderately affected group of patients and to uncover what their role in pulmonary inflammation might be.

Given the clinical difficulties in treating fungal infections, particularly in immunocompromised patients, biomarkers for disease severity and adjuvant therapies are sorely needed. The results of this study indicate that the commensal bacteria of the respiratory microbiome may be a valuable source to meet this clinical need. Though our results are highly suggestive, follow-up studies are needed to determine whether these taxonomic shifts in bacterial populations can be predictive of disease severity and mortality.

Future longitudinal studies, in which the respiratory microbiome is sampled and characterized before and/or at the beginning of *P. jirovecii* infection, could potentially identify which of these microbial markers may be prognostic markers for severe disease or fatalities, giving clinicians insight into which patients would most benefit from prophylactic treatment and closer monitoring.

This field of research would also benefit from mechanistic follow-up studies in order to uncover the role of the commensal microbiota in the context of PCP. If the differentially abundant bacteria found in this study reveal themselves to be either drivers or inhibitors of *P. jirovecii* infection, probiotic or prebiotic adjuvant therapies could be developed to mitigate disease severity. Furthermore, the variability of the respiratory microbiome between individuals suggests that personalized approaches to pulmonary diseases could be beneficial [42]. Tailoring treatments based on an individual’s unique microbiome profile could optimize therapeutic outcomes and minimize adverse effects. Personalized microbiome modulation strategies might include diet modifications, targeted antibiotics, or specific microbial supplements [43,44]. We believe that the evidence uncovered in this study indicates that there is a potential benefit to further investigating how modifications of the respiratory microbiota may help mitigate this fatal disease.

## 5. Conclusions

While there have been other publications to describe the differences in the lower respiratory microbiota between PCP(+) and PCP(−) patients, this is the first study to compare *P. jirovecii*-infected individuals with different degrees of disease severity. Though this study would benefit from a larger patient cohort, these results suggest that disease severity is indeed associated with shifts in the respiratory microbiome. Given the pressing clinical need for both biomarkers and adjuvant therapies in the context of fungal infections, particularly in the immunocompromised, we believe that this study is one of many to suggest that targeting the respiratory microbiota has important clinical potential. Furthermore, we believe that the results presented in these observational studies suggest that there is merit in designing future longitudinal ones, which may aid us in pinpointing either microbial biomarkers for disease severity or bacterial targets for therapeutic intervention in combatting these diseases.

## Figures and Tables

**Table 1:** Patient clinical characteristics. BAL: bronchoalveolar lavage. SPT: sputum.

Clinical Variables	BAL	SPT
Number of Subjects	24	9
Male	18	5
Female	6	4
Median Age	60.5	69
Underlying Pathology		
COPD	2	1
Cancer	9	4
HIV	5	2
Transplant Recipient	1	1
Other/no known pathology	7	1
Immunosuppressive Therapy	16	6
Immunodeficiency Status		
Mild Neutropenia	1	1
Moderate Neutropenia	1	0
Severe Neutropenia	2	0
Lymphopenia	9	6
Pulmonary Co-infections		
Bacterial	13	7
Fungal	9	1
SARS-CoV 2	2	1
Cytomegalovirus	1	0
None	6	1

**Table 2:** Patient clinical characteristics of mildly and severely affected patients. Mild neutropenia was defined as 1500–1000 neutrophils/µL, moderate neutropenia as 1000–500 neutrophils/µL, and severe neutropenia as less than 500 neutrophils/µL of blood. Lymphopenia was defined as less than 1000 lymphocytes/µL of blood.

Clinical Variables	Mild-Moderate	Severe
Number of Subjects	12	12
Male	9	9
Female	3	3
Median Age	58	63
Underlying Pathology		
COPD	1	1
Cancer	1	8
HIV	4	1
Transplant Recipient	1	0
Other/no known pathology	6	1
Immunosuppressive Therapy	8	8
Immunodeficiency Status		
Mild Neutropenia	1	0
Moderate Neutropenia	0	1
Severe Neutropenia	0	2
Lymphopenia	5	4
Pulmonary Co-infections		
Bacterial	6	7
Fungal	3	6
SARS-CoV 2	1	1
Cytomegalovirus	0	1
None	3	3

## Data Availability

Raw sequencing data have been deposited in the NCBI SRA database under the accession number PRJNA1137290 and will be held private until the manuscript is accepted for publication. A reviewer’s link to this data will be provided upon request.

## Supplementary Materials

The following supporting information can be downloaded at: https://www.mdpi.com/article/10.3390/pathogens14010082/s1, Table S1.

## Abbreviations

The following abbreviations are used in this manuscript:

BAL	Bronchoalveolar lavage
GM	Gut microbiota
HIV	Human immunodeficiency virus
PCP	*Pneumocystis* pneumonia
SPT	Sputum
